# HPLC-DAD Method for the Pharmacokinetic Interaction Study of Atorvastatin with Pioglitazone and Cholestyramine in Wistar Rats

**DOI:** 10.3797/scipharm.1401-18

**Published:** 2014-03-13

**Authors:** Ritesh N Sharma, Shyam S Pancholi

**Affiliations:** ^1^ Shree S. K. Patel College of Pharmaceutical Education and Research, Ganpat University, Mehsana-Gojaria Highway, Gujarat, 382014, India.; ^2^ Babria Institute of Pharmacy, Vadodara, 391240, Gujarat, India.

**Keywords:** Atorvastatin, Pioglitazone, Cholestyramine, Pharmacokinetic, Liquid-liquid Extraction, RP-HPLC-DAD

## Abstract

Carotid intima-media thickness is used as a surrogate marker for cardiovascular complications in diabetes mellitus. The combination of atorvastatin and pioglitazone was found to be effective in reducing the thickness of the carotid intima-media layer. The method of RP-HPLC coupled with a diode array detector (DAD) was developed for the pharmacokinetic interaction study of atorvastatin with pioglitazone and cholestyramine, respectively, in Wistar rats. Atorvastatin (ATR) and pioglitazone (PIO) were resolved on a C_18_ column with a mobile phase composed of 48% methanol, 19% acetonitrile, and 33% 10 mM ammonium formate (v/v/v; pH 3.5±0.3, by formic acid) and a 260 nm detection wavelength on the diode array detector. The method was validated according to international standards with good reproducibility and linear response; mean (*r*) 0.9987 and 0.9972 to ATR and PIO, respectively. The coefficients of variation of intra- and interassay precision ranged between 4.95–8.12 and 7.29–9.67, respectively. Pharmacokinetic parameters were determined in rats following an oral administration of atorvastatin in the presence and absence of pioglitazone and also with cholestyramine. Compared with the control given atorvastatin alone, the *C_max_* and *AUC* of atorvastatin were merely unchanged in rats with the co-administration of pioglitazone, while they decreased by nearly 21 and 15%, respectively, with the concurrent use of cholestyramine. There were no significant changes in *T_max_* and the plasma half-life (*T_1/2_*) of atorvastatin in both cases. The performed experiment demonstrated that the presented method was suitable for the estimation and pharmacokinetic interaction study of atorvastatin with pioglitazone and cholestyramine in Wistar rat plasma.

## Introduction

Cardiovascular complications like hypertension, atherosclerosis, and hyperlipidemia of type 2 diabetes are very common and coexisting [1]. Carotid intima-media thickness (CIMT) is increasingly used as a surrogate marker for cardiovascular complications in diabetes mellitus [2]. A reduction in the thickness of the carotid intima-media layer, which correlates with the cardiovascular risk factors and also the risk of future macrovascular events, has been observed with the use of atorvastatin (ATR) [3]. Similarly, treatment with peroxisome proliferator-activated receptor-gamma agonists, i.e. pioglitazone (PIO), have also been shown to reduce arterial pressure and carotid intima-media thickness in diabetic and non-diabetic patients at risk for cardiovascular disease [4]. The research has been done on the combination of atorvastatin and pioglitazone (PPAR gamma, agonist) and it was found to be effective in reducing the thickness of the carotid intima-media layer which correlates with the cardiovascular risk factors [5, 6].

**Fig. 1.**
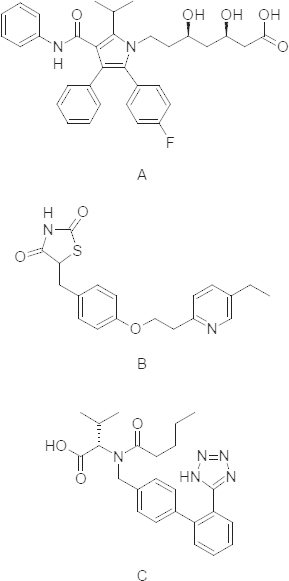
Structures of atorvastatin (A), pioglitazone (B), and Valsartan (C, ISTD)

Atorvastatin (ATR) is an inhibitor of 3-hydroxy-3-methylglutaryl-coenzyme A (HMG-CoA) reductase, which catalyzes the conversion of HMG-CoA to mevalonate, which in turn lowers the cholesterol and lipoprotein levels in the body [7]. ATR is chemically [*R*-(R*,R*)]-2-(4-fluorophenyl)-ß, δ-dihydroxy-5-(1-methylethyl)-3-phenyl-4-[(phenylamino)carbonyl]-1*H*-pyrrole-1-heptanoic acid, calcium salt (2:1) trihydrate (Fig. 1A) [8]. Atorvastatin, after oral administration in its active acidic form, is rapidly absorbed and undergoes extensive first-pass metabolism mainly by cytochrome P450 3A4 (CYP3A4) in the liver [9], which results in ortho- and para-hydroxylated derivatives and various beta-oxidation products [10]. Elimination of the drug is mainly through the bile following hepatic and/or extra-hepatic metabolism, and the mean plasma half-life of ATR is 14 hr [11].

Pioglitazone, (±)5-[[4-[2-(5-ethyl-2-pyridinyl)ethoxy]phenyl]methyl]-2,4-thiazolidinedione monohydrochloride (Fig. 1B) is a potent and highly selective agonist for the nuclear receptor peroxisome proliferator-activated receptor-gamma (PPAR-y) [12]. It decreases insulin resistance in the periphery and in the liver resulting in increased insulin-dependent glucose disposal and decreased hepatic glucose output [13].

*In vitro* pharmacokinetic studies suggest that atorvastatin is metabolized mainly by cytochrome (CYP) P450 3A4 [14]. But the *in vitro* study data revealed the involvement of multiple cytochrome isoforms in the metabolism of pioglitazone. The two main cytochrome P450 isoforms involved are CYP2C8 and to a lesser degree CYP3A4 [15]. This information suggests the likely pharmacokinetic interaction between ATR and PIO, which are metabolized by the same CYP3A4 enzyme.

Cholestyramine (CSA) is a non-absorbent anionic resin which is used to reduce the cholesterol levels in patients with hypertension by providing an opportunity for direct physical binding of bile acids and complete prevention of enterohepatic recycling of bile acids [16]. This bile acid sequestering agent is frequently prescribed with other antihypertensive drugs and the entire dose is excreted unchanged in the feces; this gives a prospect for the interaction with other co-administered drugs via direct physical interaction [17].

The literature revealed that several analytical techniques have been previously reported for atorvastatin alone in human serum by HPLC-UV [18], along with its metabolites using LC/MS in biological matrices [19]. Also, the reports are available for the analysis of pioglitazone in biological fluids including chromatographic techniques like HPLC-UV [20, 21], HPLC-MS [22], with its metabolites [23], and electroanalytical techniques like differential pulse polarography (DPP) [24] and voltammetry [25]. However, there is only one RP-LC method reported for the determination of atorvastatin calcium along with pioglitazone hydrochloride and gliquidone in human serum [26].

Looking to the above facts and considering the review of literature, we aimed to develop and validate the RP-HPLC-DAD method for the pharmacokinetic interaction study of ATR with PIO in Wistar rats. It was also decided to apply this method to determine the pharmacokinetic parameters of ATR in the presence and absence of CSA when administered alone and together in different groups of Wistar rats.

## Experimental

### Chemicals and Reagents

Atorvastatin and pioglitazone were obtained as gift samples from Acme Pharmaceutical, and Sun Pharma Ltd, Gujarat, India, respectively. Methanol, acetonitrile, and water of HPLC grade were supplied by Merck Chemicals Ltd., Ahmedabad, India. Ammonium formate and formic acid of AR grade were procured from Spectrochem Pvt. Ltd., Ahmedabad, India. Rat blood plasma was isolated from pooled blood obtained from Wistar rats.

### Apparatus and Chromatographic Conditions

The chromatographic separations were performed on the Shimadzu (Japan) HPLC system (LC-2010C_HT_) using the Gemini C18 (250 × 4.6 mm i.d, 5 um particle size) column (Phenomenex®, USA), protected with a C18 (4 × 2 mm i.d) precolumn; maintained at 35°C. The mobile phase was composed of 48% methanol, 19% acetonitrile, and 33% 10 mM ammonium formate (v/v; pH 3.5±0.2, by formic acid) which was run over 15 min at a flow rate of 1 ml/min. Analysis was performed after injecting 40 ul of the sample through an autosampler maintained at 15°C with the detection wavelength at 260 nm by using the diode array detector (DAD).

### Standard Solutions Preparation

Standard stock solutions (100 μg/mL) for ATR and PIO were prepared separately by dissolving appropriate amounts of the respective drugs in methanol. These were diluted with methanol to obtain working aqueous stocks of 10 μg/mL and 1 μg/mL (ATR only). These were mixed and volumes were made up to 10 mL in volumetric flasks to get mixed aqueous solutions of 0.06–3.0 μg/mL (ATR) and 0.2-20.0 μg/mL (PIO).

### Calibration Standard and QC Samples

Calibration standards (CS1-CS8) and QC samples representing 10% spiking were prepared in thawed plasma and 10 μL of ISTD (valsartan) aqueous solution (20 μg/mL) was added. The solutions were vortexed for 1 minute and the final concentration range 6–300 ng/mL ATR and 20-2000 ng/mL PIO in plasma was obtained. Also, three quality control samples LQC, MQC, and HQC (18, 60, and 250 ng/mL) ATR, and (60, 300, and 1500 ng/mL) PIO were prepared.

### Preparation of Rat Plasma Sample

The samples were stored in a freezer at -20°C and allowed to thaw at room temperature before processing. The extraction was performed by using the liquid-liquid extraction technique [19]. In this procedure, a 2 ml mixture of 50% ethyl acetate: 50% tertiary butyl methyl ether (TBME) (v/v) with 100 μl of 1 M formic acid (pH 3.0) were added to extract the drug from the plasma in the organic phase. After vortexing it for 3 minutes, the mixture was centrifuged at 10,000 rpm for 10 minutes. The supernatant was filtered and transferred to a glass tube to evaporate the solution under a nitrogen environment. Finally, the dried residue was reconstituted with 0.5 ml mobile phase, and transferred to 1.5-ml autosampler glass vials for RP-HPLC analysis.

### Method Validation

The developed method for the simultaneous estimation of atorvastatin calcium and pioglitazone hydrochloride was validated according to USFDA, EMEA, and ICH guidelines [27–29] for the parameters like linearity, specificity, selectivity, sensitivity, precision, accuracy, and stability.

#### Linearity

The graphs of the peak area ratios of the analytes to ISTD versus analyte concentrations in the range 6–300 ng/mL (ATR) and 20–2000 ng/mL (PIO) were plotted. The slope, *y*-intercept, and correlation coefficient were calculated for each standard curve.

#### Specificity and Selectivity

The specificity and selectivity of the method were investigated by analyzing six different lots of blank plasma and the concentration of all three analytes at the LQC level. The plasma samples were prepared as per the method described in the “Sample Preparation” section.

#### Sensitivity

Sensitivity data were evaluated by analyzing six sets of the matrix sample spiked at six for ATR, and 20 for the PIO (LLOQ) concentration, prepared as per the procedure described in the “QC Sample Preparation” section. The analytes’ calculated concentrations should be identifiable and reproducible with a precision of 20.0% and an accuracy of 80.0–120.0%. The limit of detection (LOD) and limit of quantitation (LOQ) of this method were determined by a signal-to-noise ratio.

#### Intrarun Accuracy and Precision

To establish intrarun accuracy and precision, six replicates each of LLOQ, LQC, MQC, and HQC samples of PIO and ATR were analyzed in a single analytical run on the same day and for interday, different runs on different days.

#### Extraction Recovery

The extraction efficiency of PIO, ATR, and ISTD was determined by comparing the peak areas measured after the analysis of the spiked plasma samples in triplicate at three concentrations (LQC, MQC, and HQC) with those found after direct injection of the aqueous (un-extracted) samples into the chromatographic system at the same concentration levels.

#### Stability

Sample stability in terms of short-term, process stability and freeze-thaw stability was tested by analyzing the QC samples of PIO, ATR, and ISTD at the LQC and HQC levels, while long-term stability was tested at the LQC, MQC, and HQC levels. The freeze-thaw stability (24 h at -20°C for three cycles), short-term, or bench-top stability (24 h, room temperature), and long-term stability (7 days at -20°C) were calculated. The LQC and HQC samples of drugs were stored at -15°C for 24 hr to measure the stability of the processed samples in the autosampler. The stock solution standards of PIO, ATR, and ISTD (20 μg/mL) were stored at room temperature for 24 h and 2–8°C for 7 days and the stability was evaluated after analyzing them at the ULOQ level.

### Animal Study

All animal experiments were approved (No. IAEC/2008/22) by the Shree S.K. Patel College of Pharmaceutical Education and Research Institutional Animal Ethics Committee. The Wistar rats (220±20 g) used were maintained in a clean room at a temperature between 22±2°C with 12 h light/dark cycles and a relative humidity rate of 50±5%. Rats were housed in cages with a supply of normal laboratory feed with water ad libitum. For all of the studies, the animals (n=6) were deprived of food 12 h before dosing, but had free access to water [17].

Atorvastatin calcium and pioglitazone hydrochloride suspensions for the pharmacokinetic study were prepared with normal saline using 1% Na CMC at a concentration of 5 mg/ml. Atorvastatin calcium and pioglitazone hydrochloride at a dose of 20 mg/kg were administered orally separately and together to different groups of Wistar rats. For the study of PIO in the presence of CSA, cholestyramine (CSA) was administered in the form of a fine suspension in normal saline at the concentrations of 0.115 gm/mL for the doses of 0.230 g/kg. PIO (20 mg/kg) was administered orally to Wistar rats after 0.5 hr of administration of CSA. Serial blood samples (0.25 ml) were collected from the dosed animals via the retro-orbital plexus at the designated time points, 0.25, 0.5, 1, 2, 3, 5, 8, 12, and 24 h into microtubes containing 5 μl of 5% ethylenediaminetetraacetic acid (EDTA) (20 μl/ml of blood). Plasma was harvested by centrifuging the blood using a microcentrifuge at 10000 rpm for 5 minutes and stored at -20°C until the samples of atorvastatin calcium and pioglitazone hydrochloride were analyzed by the validated RP-HPLC method.

### Pharmacokinetic and Statistical Analysis

Non-compartmental pharmacokinetic analysis was performed by using the PKSolver (a freely available menu-driven add-in program for Microsoft Excel) [30]. The area under the plasma concentration-time curve (*AUC_0-t_*) was calculated using the linear trapezoidal method. The peak plasma concentration (*C_max_*) and the time to reach the peak plasma concentration (*T_max_*) were observed values from the experimental data. The elimination rate constant (*K_el_*) was estimated by regression analysis from the slope of the line of best fit, and the half-life (*T_1/2_*) of the drug was obtained by 0.693/*K_el_*. All the mean values are reported with their standard deviation (mean ±SD). An unpaired Student’s t-test by Graph pad Prism® (ver. 5.0) was used to determine the significant difference between treatments. A value of *p* < 0.05 was considered statistically significant.

## Results and Discussion

### Method Optimization

**Fig. 2.**
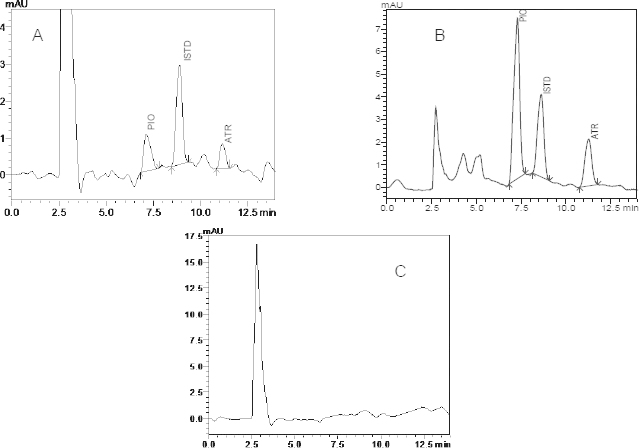
RP-HPLC chromatograms at 260 nm spiked with ISTD (valsartan) A: LQC; B: *in vivo* plasma sample obtained after 1.0 h from Wistar rats dosed with atorvastatin and pioglitazone; C: blank rat plasma

Trials were performed during optimization and some important parameters i.e. pH of the mobile phase, concentration of the acid or buffer solution, percentage and type of the organic modifier, different columns, and flow rates etc. were tested for a good chromategraphic separation of pioglitazone hydrochloride, atorvastatin calcium, and ISTD (valsartan) individually. Trials showed that the acidic mobile phase with a reversed-phase C18 column gives symmetric and sharp peaks. For this reason, 10 mM ammonium formate solution was preferred as the acidic buffer solution. Methanol was not sufficient to resolve the drug, so we introduced acetonitrile considering its high eluting power. The final mobile composition was 48% methanol, 19% acetonitrile, and 33% 10 mM ammonium formate (pH 3.5±0.2) run over 15 minute periods with a 1 ml/min flow rate. Retention times under these conditions were 7.26, 8.87, and 11.29 for PIO, ISTD, and ATR, respectively (Fig. 2). For the quantitative analytical purpose, the 260 nm wavelength was selected over the array of wavelengths obtained by the diode array detector due to decreased interferences.

Looking toward the physicochemical properties of the analytes, we attempted the liquid-liquid extraction technique to extract the ATR and PIO with its ISTD [23]. Acceptable extraction recovery and separation from the analytes prompted us to select valsartan as the internal standard. Ethyl acetate and TBME in the ratio of 50:50 (v/v) was selected over the plain ethyl acetate or TBME because of its high extraction efficiency for PIO, ATR, and ISTD. In the extracted samples, the endogenous plasma components did not produce any interfering peaks within the retention time of atorvastatin calcium, pioglitazone hydrochloride, and ISTD as shown in Figure 2.

### Method Validation

#### System Suitability

The system suitability results obtained after six replicate injections of MQC were within the 15%CV for the area ratio of PIO and ATR to ISTD. The suitability of the chromatographic system was also demonstrated by the retention time, resolution, theoretical plate, tailing factor, and capacity factor values in Table 1.

**Tab. 1.** System suitability parameters

Parameter	PIO	ISTD	ATR
Retention (*T*)	7.26	8.87	11.29
Resolution (*Rs*)	4.55	2.60	3.82
Theoretical plates (*TP*)	2356	3496	4847
Tailing factor (*Tf*)	0.95	0.97	1.21
Capacity factor (*k*')	1.40	1.93	2.73
Selectivity (α)^a^	1.38	1.41	–
Peak purity index	0.999	0.999	0.998

^a^ With respect to succeeding peak.

#### Linearity

The linearity was observed in the expected concentration range 20-2000 ng/mL (PIO) and 6–300 ng/mL (ATR) indicating its suitability for analysis. The calibration curves depict excellent correlations for PIO (mean *r*=0.9975 ± 0.001) and ATR (mean *r*=0.9967 ±0.0012) (Table 2). The back calculated % nominal concentration of the calibration standards of PIO and ATR was within the acceptable range of 88.12–109.57% and 90.41–114.32%, respectively.

**Tab. 2.** Linear regression equation data

Concentration range (ng/mL)	Slope^a^	Intercept^a^	Correlation coefficient^a^
Atorvastatin			
20–2000	0.0251 ± 0.0018, 7.11	-0.0027 ± 0.0143, -54.24	0.9967 ± 0.0012, 0.117
Pioglitazone			
50–16000	0.0097 ± 0.001, 7.27	-0.0066 ± 0.039, -33.57	0.9975 ± 0.001, 0.118

^a^ Mean ± S.D, RSD (%), n=5.

#### Specificity and Selectivity

**Fig. 3.**
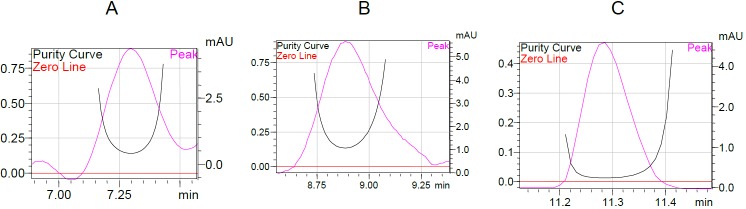
Peak purity curve of A. pioglitazone, B. internal standard, and C. atorvastatin by the diode array detector (DAD)

No endogenous source of interference was observed at the retention times of the analytes in any of the six lots of plasma when a comparison of the blank and QC level samples was made. Typical chromatograms obtained from the blank plasma and plasma samples containing the LQC of PIO and ATR are presented in Figure 2A. Also, the peak purity plots for PIO, ATR, and ISTD showed peak purity indices of nearly 1 for each and no other peaks were co-eluted with the analytes (Fig. 3).

#### Sensitivity

The RSD and % mean measured concentration representing the sensitivity at the LLOQ level was found to be 7.29 and 105.75 for ATR and 6.02 and 98.75 for ATR, respectively. The lowest concentrations of 10 ng/mL (PIO) and 6 ng/mL (ATR) were selected as the LOQ. The LOD measured for this method was 2 and 3 ng mL^-1^ for PIO and ATR, respectively.

**Tab. 3.** Intraday and interday precision and accuracy data

Intraday precision and accuracy				Interday precision and accuracy		
Nominal conc., (ng/mL)	Concentration measured	% RSD	Accuracy (%)	Concentration Measured	% RSD	Accuracy (%)
			Atorvastatin			
6	6.41±0.5	8.12	105.11±4.1	6.30±0.6	9.67	106.86±6.3
18	18.42±1.3	7.05	102.34±3.5	18.63±1.4	7.29	103.51±7.3
60	60.78±6.3	6.26	101.30±4.2	63.64±5.3	8.36	106.08±3.9
250	255.21±16.8	6.58	102.08±3.3	267.35±20.4	7.63	106.94±6.1
			Pioglitazone			
20	19.54±1.3	6.53	98.89±3.1	21.12±2.1	9.51	105.59±5.1
60	59.77±3.3	5.53	99.62±2.4	61.64±5.1	8.15	102.75±4.8
300	298.62±21.5	5.42	99.16±3.5	296.81±32.7	8.21	99.71±4.6
1500	1486.71±73.9	4.95	99.11±4.2	1579.73±129.2	8.18	105.32±3.9

^a^ Mean ± S.D, n=6.

#### Intra- and Interday Accuracy and Precision

The accuracy and precision from the plasma samples were suggested by means of the concentration found and the RSD, respectively, at four QC samples (20, 60, 600, and 1500 ng mL^-1^) of PIO and (6, 18, 60, and 250 ng/mL) of ATR (Table 3). The mean concentrations for the intrarun accuracy were 98.8±3.1–99.6±4.2% (PIO) and 101.30±3.5–105.1±4.2% (ATR). The developed method was found to be precise as the RSD values for the intrarun precision study were 4.92-6.53 (PIO) and 6.26-8.12 (ATR). The interrun accuracy of the six replicates’ analysis of the four QC samples of PIO and ATR was 99.7±4.6–105.5±5.1% and 103.5±7.3–106.9±3.9%, respectively. The RSD values for the interrun precision study were 8.15–9.51 (PIO) and 7.29–9.73 (ATR).

#### Extraction Recovery

The mean % recoveries with %CV across three concentrations were 74.03±2.5% (ATR) and 70.52±2.8% (PIO); they suggested a good extraction efficiency of the method (Table 4). Also, the mean % recovery with %CV of the internal standard was also found to be 68.73±6.1% within an acceptable range.

**Tab. 4.** Extraction recovery data from rat plasma

Analyte (s)	Nominal conc., (ng/mL)	Recovery (%)	%RSD^a^
Atrovastatin	18	72.11	8.61
	60	74.14	7.90
	250	75.86	8.23
	Across mean	74.03	2.54

Pioglitazone	60	68.94	5.17
	300	69.84	7.72
	1500	72.78	4.72
	Across mean	70.52	2.85

ISTD	200	65.74	6.07

^a^ n=6

#### Stability

The stability tests of the analytes were designed to cover the expected conditions of handling clinical or preclinical samples (Table 5). No significant loss of the analytes in the stock solution was observed when stored at room temperature and 2-8ºC, respectively and analyzed at the ULOQ level. The stock solutions of PIO, ATR, and ISTD were stable for at least 7 days and the mean % change was less than 10 %. Samples during the withdrawal from the freezer and their preparation were found to be stable with a % mean concentration change of -0.89 (LQC) and -0.34 (HQC) for PIO and -1.60 (LQC) and -1.28 (HQC) for ATR. The extracted samples of the drugs were stable at 15°C for at least 24 hr in the autosampler. The mean % change of concentration values at the LQC and HQC level after the third cycle of freeze-thaw stability studies were -1.02 and -0.73 (PIO), and -1.16 and -0.96 (ATR). The QC samples were stable at -20°C for 7 days (long-term); the results showed no significant loss of the analytes. The final stability test was demonstrated on the plasma and the mobile phase used in the study, and no significant deterioration of the analytes was observed.

### Pharmacokinetic Interaction Study of Atorvastatin Calcium in the Presence and Absence of Pioglitazone Hydrochloride and Cholestyramine

The validated RP-HPLC method was applied for the estimation of atorvastatin calcium and pioglitazone hydrochloride in rat plasma samples, which were administered to rats alone and together in Wistar rats (Fig. 2B). The pharmacokinetic parameters, i.e. *AUC_(0-t)_*, *C_max_*, and *T_max_* etc. (Table 6), were calculated from the mean plasma concentration-time profile (Fig. 4) for ATR and PIO as the control and also ATR in the presence of PIO and CSA.

The results of the ATR and PIO study, parameters *AUC_(0-t)_* and *C_max_* when ATR as the control was administered alone to the group of Wistar rats, were 884± 98 ng.h/mL and 94.4±27.9 ng/mL, respectively. But when ATR was co-administered with PIO to the Wistar rats, *AUC_(0-t)_* and *C_max_* were 879± 92 and 89.7±27.3, respectively. The results suggested that *AUC_(0-t)_* and *C_max_* of ATR in the presence of PIO were not significantly changed and also there was no change observed in *T_max_*, *T_1/2_*, and *K_el_* parameters. The effect of atorvastatin on the pharmacokinetics of pioglitazone was also observed by determining the pharmacokinetic parameters for pioglitazone alone and also concomitantly with ATR (Fig. 4C). The parameters *AUC_(0-t)_* and *C_max_* for PIO alone were 8060±1073 and 1064.0±372.9, respectively, and after being co-administered with ATR, were found to be nearly the same as 7817±938 and 981.6±354.6, respectively, with no change in *T_max_* and *T_1/2_*.

**Tab. 5.** Stability data for atorvastatin and pioglitazone

Storage Condition	Analytes	Conc. (ng/ml)	Concentration measured %)^a^	
			Fresh	Stability
Main stock solution stability at RT^b^ for 24 hr	ATR	300	101.39±2.0	100.42±2.4
	PIO	2000	101.41±2.8	99.97±3.1
	ISTD	2000	101.22±1.4	100.34±1.9

Main stock solution stability at 2–8°C for 7 days	ATR	300	100.32±0.9	99.72±1.9
	PIO	2000	101.82±2.4	100.67±2.7
	ISTD	2000	100.35±1.3	99.75±1.8

Benchtop stability at RT for 24 hr	ATR	18	101.43±6.7	99.82±7.2
		250	108.99±5.9	107.60±6.2
	PIO	60	100.75±6.7	99.86±7.1
		1500	101.45±2.8	101.09±6.7

Process stability at 15°C for 24 hr	ATR	18	100.16±6.2	99.88±6.4
		250	103.75±6.1	103.27±7.1
	PIO	60	101.53±4.8	101.12±8.2
		1500	108.89±6.0	108.54±7.8

Freeze-thaw at -20°C for 24 hr (three cycles)	ATR	18	101.82±6.2	100.62±6.3
		250	101.77±6.5	100.74±7.3
	PIO	60	101.95±7.1	100.90±8.5
		1500	107.76±5.8	106.97±7.7

Long-term stability at -20°C for 7 days	ATR	18	103.23±5.7	102.22±6.2
		60	104.95±8.0	103.60±8.5
		250	106.92±6.6	105.81±7.3
	PIO	60	101.46±6.9	101.09±8.5
		300	111.42±3.5	110.34±8.3
		1500	104.74±7.5	104.07±8.3

^a^ Mean ± S.D, n=6;

^b^ room temperature.

The pharmacokinetic parameters, i.e. *AUC_(0-t)_, C_max_, T_max_*, and *T_1/2_*, were also calculated from the mean plasma concentration-time profile (Fig. 4B) for ATR in the presence of cholestyramine (CSA). The results revealed that *AUC_(0-t)_* and *C_max_* of atorvastatin calcium in the presence of CSA were 756±79 ng.h/mL and 74.6±22.1 ng/mL, respectively. The results suggested that *AUC_(0-t)_* of atorvastatin calcium was reduced nearly by 15±1% and also *C_max_* decreased by 21±1 % with no significant effect on *T_max_* and *T_1/2_*.

Statistical significance was calculated by performing two-tailed unpaired Student’s t-test between groups like 1) ATR and ATR-PIO, 2) ATR and ATR-CSA, and 3) PIO and PIO-ATR and the *p*-values for the groups were found to be 0.941, 0.475, and 0.878, respectively. As the value of *p* <0.05 was considered statistically significant; so all the pharmacokinetic interactions between them were not statistically significant. But if we compare the *p*-values between the different groups; the *p*-values for the groups ATR and ATR-CSA were merely half, showing some change in pharmacokinetics.

The co-administration of ATR with PIO resulted in no change in metabolism of ATR as unveiled from the pharmacokinetic study results. Also, there was no change observed in the metabolism of PIO. Although both drugs are substrates of CYP 3A4 and metabolised through it significantly, they do not affect each other’s metabolism as assumed by us. Elimination of both of the drugs was also almost the same as revealed by the results. On the other hand, a decrease in the AUC_(0-t)_ and C_max_ values were observed for ATR coadministered with CSA as compare to the respective control in Wistar rats, proving the concept of physical interaction. Since atorvastatin does not undergo hepatobiliary circulation, this might be the reason for the insignificant interaction with CSA.

**Tab. 6.** Mean pharmacokinetic parameters of atorvastatin in the absence and presence of pioglitazone (PIO) and cholestyramine (CSA) in Wistar rats

Parameter	Atorvastatin (Control)	Atorvastatin (PIO)	Atorvastatin (CSA)	Pioglitazone (Control)	Pioglitazone (ATR)
*AUC_(0-t)_ (ng.h/mL)*	884±98	879±92	756±79	8060±1073	7817±938
*AUC_(0-∞)_ (ng.h/mL)*	1005±138	1003±127	860±83	8427±1121	8155±1035
*C_max_(ng/mL)*	94.4±27.9	89.7±27.3	74.6±22.1	1064.0±372.9	981.6±354.6
*T_max_ (h)*	2.0	2.0	2.0	3.0	3.0
*T_1/2_*	6.23±1.92	6.69±2.18	7.04±2.13	4.78±2.34	5.09±3.05
*K_el_ (h^-^)*	0.119±0.038	0.111±0.037	0.105±0.032	0.124±0.039	0.132±0.042

Data are derived from the mean of six animals and are shown as the mean± SD.

All the data points are not statistically different with respect to the control (*p* < 0.05).

## Conclusion

In the present work, a simple, rapid, and sensitive reversed-phase HPLC method has been developed and validated for the estimation of ATR and PIO in rat plasma using a DAD detector. The validated method was also found to be accurate, precise, and specific. This method was successfully used to determine the pharmacokinetic parameters of ATR and PIO administered alone and together to different groups of Wistar rats. The co-administration of ATR in the presence and absence of PIO resulted in no change in metabolism. And physical interaction with CSA resulted in a low plasma concentration as evident from the results. All the pharmacokinetic parameters depicted are not significantly different from their respective control group, so there are no specific pharmacokinetic interactions which seem to be observed between them. The method presented can be used for the estimation of analytes in pharmaceutical formulations and also for their bioavailability and bioequivalence study in humans.
